# Multi-Agent Sensor Fusion Methodology Using Deep Reinforcement Learning: Vehicle Sensors to Localization

**DOI:** 10.3390/s26041105

**Published:** 2026-02-08

**Authors:** Túlio Oliveira Araújo, Marcio Lobo Netto, João Francisco Justo

**Affiliations:** Sistemas Eletrônicos, Programa de Pós-Graduação em Engenharia Elétrica, Escola Politécnica da Universidade de São Paulo, São Paulo 05508-010, Brazil; marcio.netto@usp.br (M.L.N.); joao.justo@usp.br (J.F.J.)

**Keywords:** autonomous vehicles, carla simulator, deep reinforcement learning, urban vehicle simulation

## Abstract

Despite recent major advances in autonomous driving, several challenges remain. Even with modern advanced sensors and processing systems, vehicles are still unable to detect all possible obstacles present in complex urban settings and under diverse environmental conditions. Consequently, numerous studies have investigated artificial intelligence methods to improve vehicle perception capabilities. This paper presents a new methodology using a framework named CarAware, which fuses multiple types of sensor data to predict vehicle positions using Deep Reinforcement Learning (DRL). Unlike traditional DRL applications centered on control, this approach focuses on perception. As a case study, the PPO algorithm was used to train and evaluate the effectiveness of this methodology.

## 1. Introduction

Autonomous transportation has attracted significant attention in recent years from both academia and industry [1]. Technologies such as self-driving, 5G, edge/cloud analytics, artificial intelligence, and augmented/virtual reality are shaping future vehicles. Despite recent developments in sensing and processing hardware, fully reliable autonomous vehicles remain elusive, as evidenced by recent accidents involving semi-autonomous vehicles [2]. Particularly in complex traffic scenarios, sensor-based automated decision efficiency is limited, requiring advanced artificial intelligence (AI) solutions—for instance, for lane merging [3], highway platooning [4], and roundabout navigation [5].

Recent studies have shown that data exchange between vehicles, infrastructure, and the cloud (V2V, V2I, and V2C, respectively) can significantly enhance perception by enabling accurate detection and identification of the environment. Using probabilistic and neural networks, it is possible to identify and classify objects, building a comprehensive navigation map with information that can improve perception for all connected vehicles [6]. Deep reinforcement learning (DRL) is a machine learning technique that has been leveraged for several traffic and cooperative control tasks, such as DRL-based traffic signal control for emission reduction in cooperative vehicle-infrastructure systems [7], and a cooperative DRL-based control model for CAV and traffic signaling proposed in [8]. This paper introduces a novel approach to creating a shared online map/database, integrating sensor data from vehicles with a DRL agent, simulating a V2C scenario. The agent employs DRL to fuse multimodal sensor data and infer the positions of all vehicles. Unlike conventional DRL applications in autonomous vehicles, which focus on control, this work emphasizes perception and sensor fusion. Several case studies were conducted to evaluate the efficiency of this approach in accurately localizing all vehicles in multi-sensor scenarios.

## 2. Connected and Autonomous Vehicles Background

### 2.1. Vehicle Sensors

**Figure 1:**
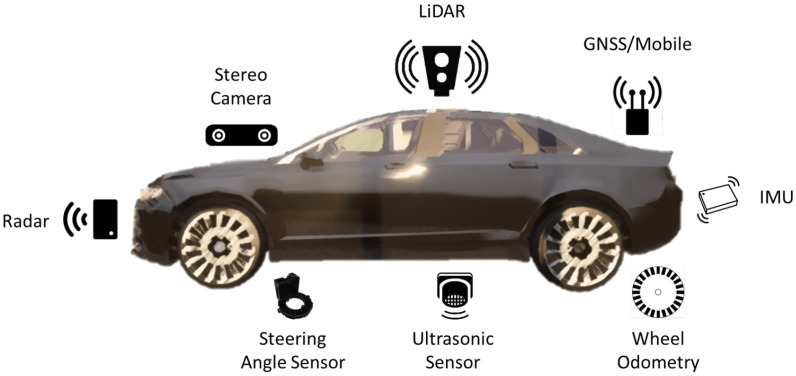
Autonomous Vehicle Sensors.

Sensors are essential for vehicle perception systems. Understanding their functions and the information they provide is crucial for designing autonomous vehicles and their data processing strategies. The most commonly used sensors are ultrasonic, radar, cameras, LiDAR, GNSS, IMU, steering angle sensors, and wheel odometry [9]. Multiple sensor types can be found within a single autonomous vehicle (AV), as illustrated in Figure 1.

To detect objects in the vehicle’s path, it is desirable to employ a combination of sensors, allowing each to compensate for possible limitations of the others. A comparison of the main features of the sensors most commonly used for AVs is provided in Table 1, adapted from [10,11,12]. Within the CarAware framework [13], all sensors mentioned in this section are implemented and ready for application in any training scenario. Some of these sensors include onboard pre-processing and are considered smart sensors.

### 2.2. Vehicular Connectivity

Even with state-of-the-art sensors and computational capabilities, autonomous vehicles face challenges in object detection, recognition, and situational awareness, especially due to environmental effects, operational regions, field-of-view occlusions, unpredictable motion, and other events. Each vehicle’s sensing area is inherently limited. Connecting an AV (thus forming a CAV) allows for the extension and enrichment of available information, based on the data of other vehicles and objects.

To enable this, certain connectivity technologies must be present in both vehicles and infrastructure. The main concepts include:

- 5G: The fifth generation of mobile networks, offering data transfer speeds from 1 to 10 Gbps with latencies as low as 1 ms [14], thus supporting real-time applications.
- Cloud computing: All connected devices leveraging online processing and storage resources.
- V2X: Encompasses all vehicle communications with “anything” (vehicle-to-infrastructure, vehicle-to-vehicle, vehicle-to-device, vehicle-to-grid, and vehicle-to-cloud).

For interoperability, common standards are required. Thus, the SAE established standards defining message formats and protocols, such as the Basic Safety Message (BSM, SAE J2735) [15]. BSM is a broadcast message, sent up to ten times per second with each vehicle’s basic data (e.g., latitude, longitude, elevation, speed, and heading).

### 2.3. Carla Simulator and CarAware Framework

CARLA is an open-source simulator for urban driving [16]. It is categorized as nanoscopic, but supports microscopic simulations as well. Its strengths include realistic vehicle and pedestrian dynamics, detailed urban environments and graphics, the ability to script scenarios, and support for a broad range of sensors.

**Figure 2:**
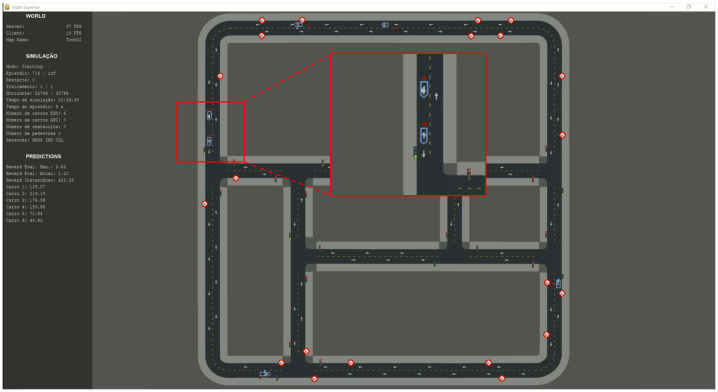
Case study predictions after curriculum training. Red dots represent predicted positions, and blue arrows represent the true positions.

Due to CARLA’s open and highly customizable nature—and the absence of manufacturer-provided user interfaces for rapid experiments—a new framework was developed: CarAware [13]. CarAware runs atop CARLA, harnessing its 3D simulation capabilities while adding a simplified top-down visualization and a HUD showing critical training metrics. Figure 2 shows the main interface. Code for both the simulation framework and PPO implementation is available at https://github.com/tulioaraujoMG/CarAware (accessed on 27 December 2025).

## 3. Deep Reinforcement Learning Background

### 3.1. Overview

Reinforcement Learning (RL) describes how animals (and, now, machines) learn by interacting with their environment through observations and actions, receiving rewards or penalties and reinforcing desired behaviors. This principle is central to machine learning: agents interact with their environment via sensors and actuators, and their actions are evaluated by a reward function. Based on the reward, the agent learns which actions maximize cumulative reward—a process analogous to human learning. This technique is implemented in the framework to undertake the training for the proposed case study.

Advances in deep learning have transformed RL by leveraging neural networks to estimate the value of observation/action pairs (value-based methods) or to directly estimate the best decision policies (policy-based methods). As shown in [17], DRL algorithms are widely applied in areas such as robotics, autonomous control, communications, natural language processing (NLP), games, and computer vision. Tools exist to interpret the intrinsic “black box” behaviors of DRL models and validate their safety and efficiency [18].

With increased computational power, deep RL (DRL) methods have emerged, utilizing neural networks to approximate the large tables otherwise required for mapping state-action or state-value pairs.

**Figure 3:**
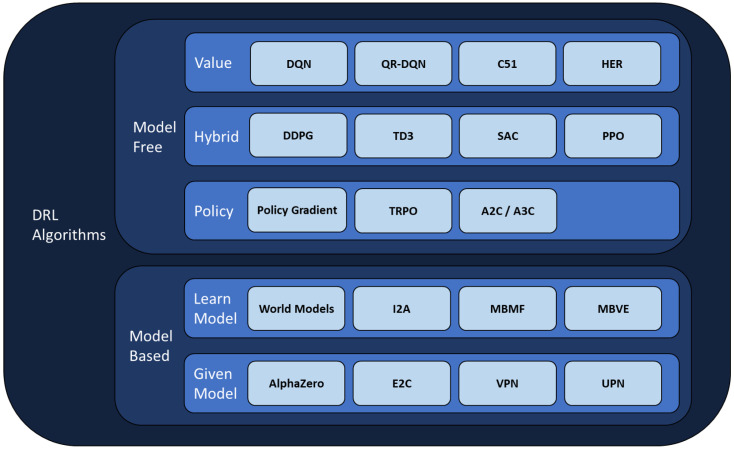
Main DRL algorithms and their classification (Adapted from [19]).

According to OpenAI [19], DRL algorithms can be classified as depicted in Figure 3. The highest distinction is between model-free algorithms, which learn by directly interacting with the environment, and model-based algorithms, which learn or are given a model of the environment to facilitate planning [20].

Model-free algorithms fall into two main types: value-based (e.g., Q-Learning) and policy-based (e.g., Policy Optimization). Value-based algorithms focus on learning a value function that finds the most rewarding states and actions; policy-based algorithms directly learn the policy that yields the best rewards for each state. All DRL algorithms have a policy, but the focus of training differs: value-based on state/action values; policy-based on directly optimizing the policy.

In policy-based methods, rather than learning the value function, the policy is optimized directly to increase the likelihood of favorable outcomes. These methods can learn stochastic policies (probabilistic outputs), in contrast to the deterministic policies typically found in value-based approaches.

Some methods, such as actor–critic, combine both paradigms: the “actor” chooses actions according to the current policy (policy-based), while the “critic” evaluates these actions (value-based), providing feedback to improve performance.

PPO (Proximal Policy Optimization) [21] is a hybrid policy-based/value-based DRL algorithm, well-suited for continuous observation and action spaces. In PPO, actor and critic networks improve training stability by constraining policy updates, avoiding overly large and possibly disruptive changes. PPO is efficient, easy to implement, and robust for diverse applications—a justification for its use in this work.

PPO applies incremental updates to the actor network, calculating the ratio between the current and previous policy probabilities using Equation (1),

(1) $$r_{t}\left(\theta \right)=\frac{\pi_{\theta }\left(a_{t}|s_{t}\right)}{\pi_{\theta_{old}}\left(a_{t}|s_{t}\right)}$$

where $\pi_{\theta }$ is the probability of taking an action $a_{t}$ at state $s_{t}$ in the current policy, and $\pi_{\theta \mathrm{old}}$ in the previous one. If $r_{t}\left(\theta \right)$ is greater than 1, the current policy is more likely to select action $a_{t}$ at state $s_{t}$; if between 0 and 1, the previous policy was more likely. The advantage function, typically estimated through GAE (Generalized Advantage Estimation), quantifies how much better a particular action performs relative to average, as in Equation (2),

(2) $$\begin{array}{cc} & \delta_{t}=r_{t}+\gamma V\left(s_{t+1}\right)-V\left(s_{t}\right)\\ & {\hat{A}}_{t}=\delta_{t}+\left(\gamma \lambda \right)\delta_{t+1}+\ldots +{\left(\gamma \lambda \right)}^{T-t+1}\delta_{T-1} \end{array}$$

where λ is an exponential mean discount factor, γ is the trajectory discount, and $\delta_{t}$ is the TD advantage estimate.

### 3.2. Curriculum Learning

**Figure 4:**
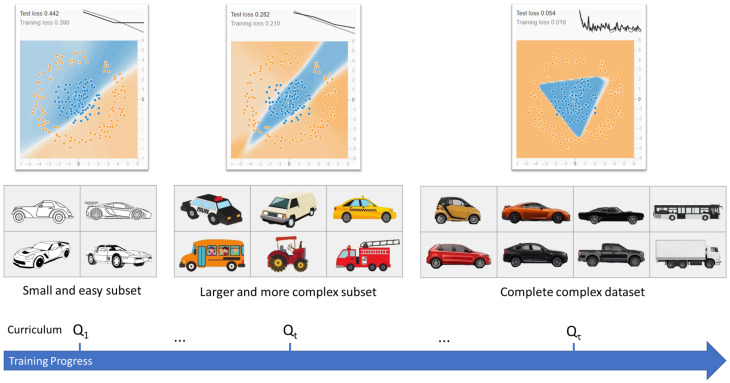
Example of a curriculum-learning process for computer vision.

Curriculum learning (CL) is a training strategy that enhances an agent’s generalization and convergence by presenting a sequence of tasks of gradually increasing difficulty [22]. Figure 4 shows an example. In DRL and other machine learning paradigms, CL helps guide agent learning, particularly in complex settings: agents first learn from simple environments and then progressively face harder ones, thus fostering better generalization.

A common approach is to start with simple training scenarios and gradually present more complex ones, improving the agent’s ability to link observations and outputs. This methodology has succeeded in not only DRL, but also unsupervised and supervised learning applications, including computer vision, NLP, and autonomous vehicle control [23].

## 4. Collective Perception Methodology

### 4.1. Training Setup

**Figure 5:**
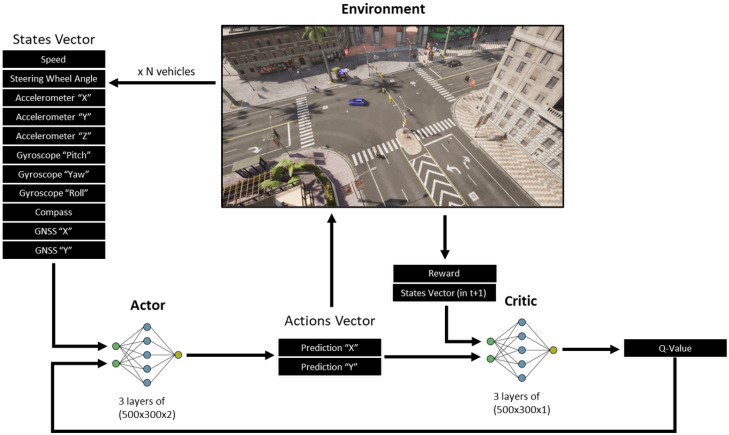
Complete implemented training architecture.

The DRL training was performed in a single environment within the CARLA simulator, using the CarAware framework. To deploy the PPO algorithm, an actor–critic architecture was implemented, comprising an actor network $\pi (a_{t}|s_{t};\theta )$ and a critic network $V(s_{t};\theta_{v})$, both instantiated as distinct MLPs (as the input $s_{t}$ is a vector). As shown in Figure 5, their configurations are as follows:

- *Actor Network*: An MLP with three fully connected layers of dimensions 500×300×2, where 500×300 defines the hidden layers (selected experimentally) and 2 is the action output dimension (coordinates *x*, *y*). The first two layers use ReLU activation; the last uses no activation. Input and output are normalized to [−1, 1] to prevent early weight overfitting. During training, actions are drawn from the multivariate Gaussian $a_{i}∼N\left(\mu_{i},\sigma_{i}\right)$; during evaluation, the mean $\mu_{i}$ is directly used.
- *Critic Network*: An MLP with three fully connected layers of dimensions 500×300×1, where 1 is the output (value function $V\left(s_{t};\theta_{v}\right)∼R\left(s_{t}\right)$). The first two layers use ReLU activation; the last uses none.

The actor network outputs (mean and standard deviation) are used to calculate the policy change ratio (Equation (1)), necessary for the Clipped Surrogate Objective Loss (CSOL, Equation (3)):

(3) $$L_{t}^{CSOL}\left(\theta \right)=\epsilon_{t}\left[L_{t}^{CLIP}\left(\theta \right)-c_{1}L_{t}^{VF}\left(\theta \right)+c_{2}S\left[\pi_{\theta }\right]\left(s_{t}\right)\right]$$

The DRL PPO training process consists of:

1. Start a simulation episode, spawning vehicles and assigning automatic agents.
2. Store tuples $\left(s_{t},a_{t},r_{t},V\left(s_{t};\theta_{v}\right)\right)$ for each transition.
3. Calculate advantage estimates using GAE (Equation (2)).
4. Divide the complete horizon of data into stochastically sampled mini-batches, and feed data into the actor–critic network, defined by the hyperparameter “Epoch Number”. The training process optimizes the parameters of actor (θ) and critic ($\theta_{v}$) networks via Adam optimizer based on the Clipped Surrogate Objective Loss ($L^{CSOL}$) function.
5. Run the previous steps repeatedly until all episodes have been completed (define in “Episodes Number”).

### 4.2. Training Methodology

To showcase the framework’s capabilities, a series of DRL case studies was carried out. The objective was to use input data from GNSS, IMU, and SAS/WO sensors in multiple simulated vehicles to accurately infer the position of every vehicle from the top-view window. These sensors are standard in CAVs, supplying motion and position data (used in BSM protocol messages). The choice is further justified by their output bandwidth, which is far lower than that of complex sensors like cameras, radar, and LiDAR—minimizing computational demands for this proof-of-concept. Sensor frequencies: GNSS at 1 Hz, IMU at 100 Hz, SAS/WO at 10 Hz; all sensor outputs are affected by scenario-varying standard deviation (noise, low = 0.00001 and high = 0.0001), but without bias. Simulated blackout events occur every 5–10 s, with a duration of 5–10 s (randomly defined).

The selected algorithm for this application was Proximal Policy Optimization (PPO), which has proven effective for continuous input/output DRL problems in complex domains [21]. Each vehicle’s observation comprises a nine-element vector (GNSS “X” and “Y”; IMU: accelerometer X,Y,Z, gyroscope pitch, yaw, roll, compass; and SAS steering angle and WO speed), and the action space is a two-element vector representing the agent’s prediction of the x,y coordinates. The DRL agent acts on each vehicle individually per training step, cycling through all vehicles, simulating a V2C setup. The reward function is the Euclidean distance between predicted and real positions (simulator ground truth). Hyperparameters are shown in Table 2.

It is important to emphasize that the primary focus of this work is on perception, with the expected output being an accurate estimation of vehicle positions. This methodology differs from the regular DRL scenario since the vehicles’ actions (localization estimations) do not cause direct impacts on the environment. While the reward is directly affected by each predicted action, this characteristic does not pose a problem for the training scenarios considered. Deep Reinforcement Learning (DRL) was selected over other training methods—such as supervised regression or Bayesian filtering—because of its strength in learning from heterogeneous input types, without the need for explicit understanding or conversion of sensor data into a unified format. Moreover, this approach allows for the straightforward integration of additional sensor types in future work.

## 5. Results and Discussions

### 5.1. Scenario 1: Town 02—Localization with All Sensors and No Blackout Events

The first task was to train an agent to infer the positions of all vehicles in the map using BSM-associated sensors. The scenario assumed perfect communication and continuous sensor data availability. The “Town 02” map was selected for its urban features and compact size.

**Figure 6:**
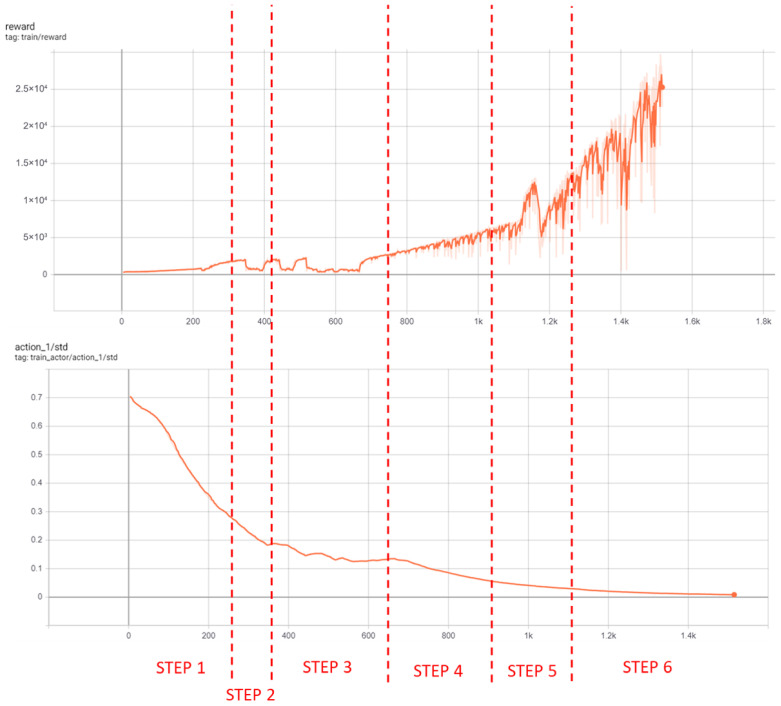
Reward and standard deviation curves for scenario 1.

In high-complexity settings like urban driving, lower learning rates improve convergence by allowing more input analysis per update. Adding input noise fosters better generalization. Initial attempts showed the agent could not consistently achieve multi-agent position prediction through one-step training, even after hyperparameter optimization, due to environmental complexity. Curriculum Learning (CL) was necessary. Six curriculum steps were adopted (see Table 3), with training evolution shown in Figure 6.

After 109 h of training over 1510 episodes, the agent demonstrated robust learning capacity and was able to accurately locate vehicles in the tested scenario (see Figure 2). However, the agent’s predictions were less accurate in less frequently explored map regions (e.g., roads in the middle). As suggested by DRL theory, asynchronous training with parallel environments training would further improve generalization, but was beyond available computational resources and time.

### 5.2. Scenario 2: Town 02—Localization with Eventual GNSS Blackout Events

In this scenario, the goal was to enable the agent to cope with temporary GNSS sensor failures, localizing vehicles using only the remaining sensors. Since GNSS directly provides (x,y) coordinates, its unavailability presents a major challenge. Complete loss makes localization impossible, but short outages may be mitigated by other sensor data.

**Figure 7:**
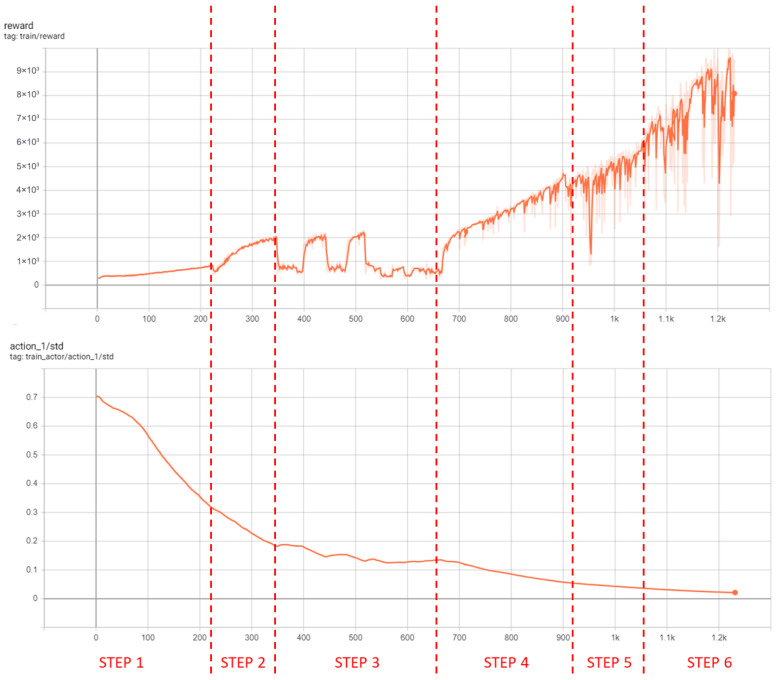
Reward and standard deviation curves for scenario 2.

Transferring an agent trained only on reliable GNSS to this new scenario did not work: without new training, the agent simply kept the last observed GNSS output. Continuing training from the previous agent produced unsatisfactory results (the output had low entropy and poor adaptation to the new challenge). Therefore, curriculum learning was again needed, training the agent from scratch and introducing GNSS outages partway through training. Results are shown in Table 4 and Figure 7.

**Figure 8:**
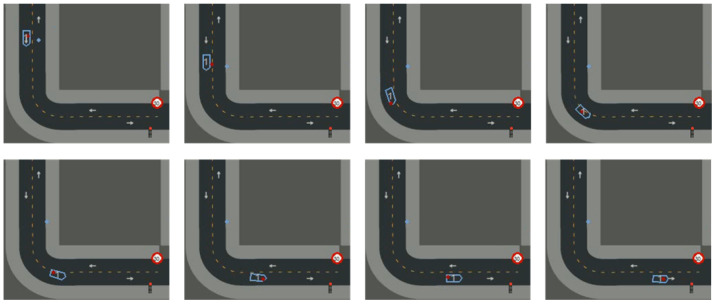
Correct prediction under GNSS blackout event.

**Figure 9:**
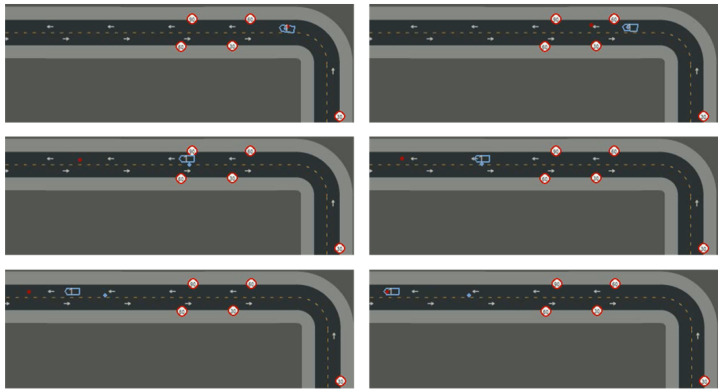
Wrong behaviors learned by the agent to deal with GNSS blackout events.

After 87 h of training (1230 episodes), results showed that the agent could maintain accurate predictions for up to 10 s of GNSS blackout—including during curves (Figure 8). Occasionally, the agent learned to offset predictions ahead of the vehicle position (Figure 9). This undesirable compensation illustrates the challenge of avoiding local optima in DRL.

### 5.3. Scenario 3: Town 02—Localization with Eventual IMU, SAS/WO Blackout Events

Here, the objective was to predict vehicle positions during IMU and SAS/WO sensor outages, simulated via random blackout events (that could happen at the same time or not). An agent initialized from previous phases (before GNSS blackout bias) was used.

**Figure 10:**
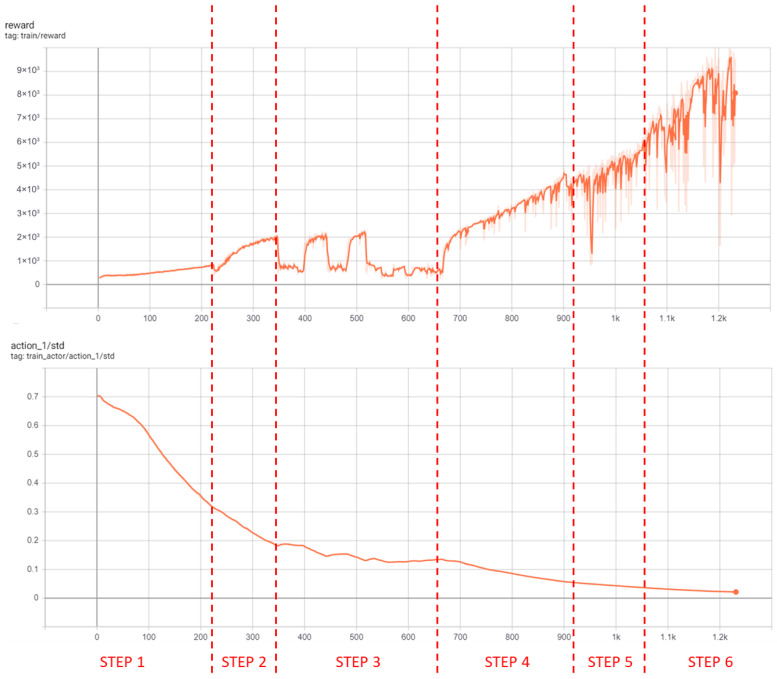
Reward and standard deviation curves for scenario 3.

Curriculum learning was again applied (see Table 5 and Figure 10). With the GNSS sensor remaining available, predictions remained accurate, with the agent learning to increase the weight placed on GNSS input during blackouts.

**Figure 11:**
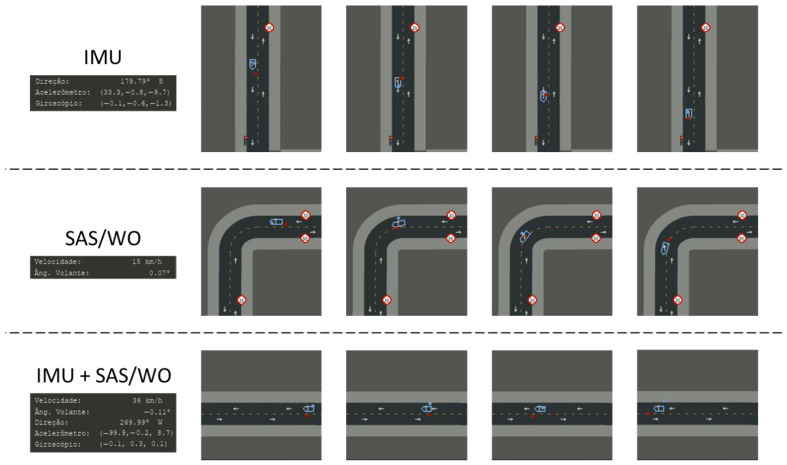
Correct prediction under IMU and SAS/WO blackout events.

After 79 h of training (1310 episodes), the agent was able to maintain accurate localization during temporary IMU/SAS/WO outages (see Figure 11). This outcome was anticipated, as the GNSS sensor serves as the primary source of localization information and therefore contributes most significantly to vehicle position prediction compared to the other sensors. When multiple sensor blackouts occurred, the agent learned to increase the reliance on GNSS data within its decision-making process.

### 5.4. Scenario 4: Town 01—Localization with All Sensors and No Blackout Events

**Figure 12:**
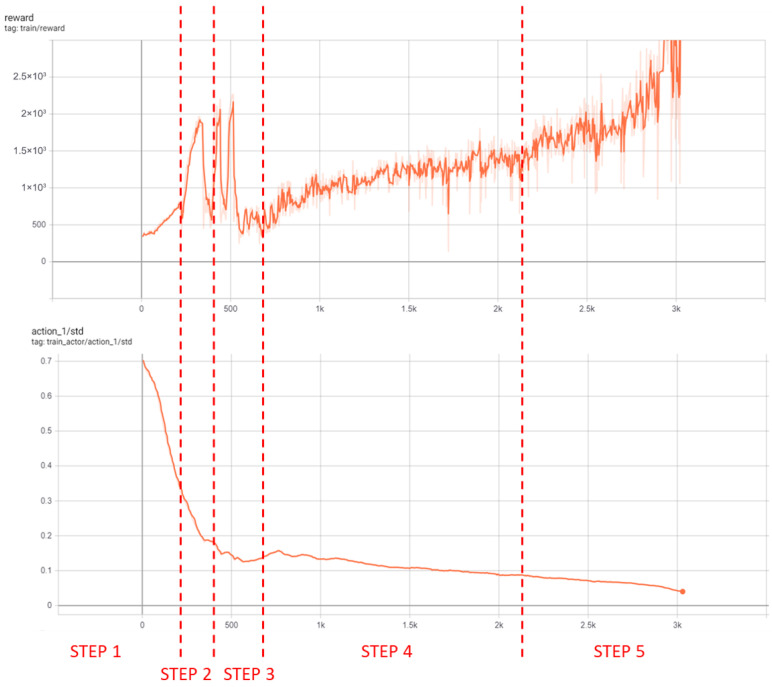
Reward and standard deviation curves for scenario 4.

Similar to scenario 1, this simulation was conducted to evaluate both the training methodology and the agent’s performance on a different map, aiming to verify whether the conclusions remained consistent regardless of the training environment. “Town 01” was chosen as it also offers a realistic urban setting and, although larger than “Town 02”, remains sufficiently compact to ensure that computational resources were not a limiting factor. The same assumptions and conditions applied as in the previous scenarios. The curriculum (Table 6) and result curves (Figure 12) follow the same structure.

After 210 h (3003 episodes), localization was achieved, but with increased difficulty and some areas showing reduced model efficiency—attributable to higher map complexity and computational cost. The previously noted challenges regarding the DRL methodology and computational resource limitations are also present in this scenario; however, they are even more pronounced here due to the larger map size, which introduces additional complexity and a greater number of details for the agent to process and learn in order to make accurate predictions.

## 6. Conclusions

Simulating urban autonomous vehicles is computationally intensive. A downside of DRL training is that it requires substantial time for the model to learn, compared to other machine learning techniques, since it always starts from a “blank slate” state.

Both positive and negative aspects were observed in the adopted training strategies. The trained model was able to partially fulfill the intended objective, although some undesired behaviors were present. Certain limitations, such as the available training time and computational resources, impacted model performance. Nevertheless, results were sufficient to confirm that the proposed methodology is feasible for real-world applications.

Applying asynchronous training with multiple environments and diverse maps—allowing mixed experience to be gathered for each training step—can considerably improve PPO learning, reducing biases and strengthening generalization [21,24]. A common challenge in reinforcement learning is a lack of generalization capability [25]. Recent studies suggest that blending learning approaches, for example, integrating imitation learning with DRL, can improve generalization [26]. Moreover, simulating more dynamic traffic events and complex interactions between vehicles, and implementing more complex reward functions, like multi-objective rewards that also penalize prediction uncertainty (e.g., negative log-likelihood of the Gaussian output) and favor trajectory smoothness (e.g., second-order difference in successive estimates) could improve training effectiveness.

With further improvements, these methodologies could be deployed in real backend systems, collecting vehicle data and providing information to increase the situational awareness and safety of connected and autonomous vehicles in real traffic.

## Figures and Tables

**Table 1:** Comparison of sensor features (Adapted from [10,11,12]).

Feature	Ultrasonic	RADAR	LiDAR	Camera
Primary Technology	Sound wave	Radio wave	Laser beam	Light
Range	∼5 m	∼250 m	∼200 m	∼200 m
Infrared Frequency	40–70 kHz	24, 74 or 79 GHz	193 or 331 THz	272–1498 THz
Affected by Weather	Yes	No	Yes	Yes
Affected by Lighting	No	No	No	Yes
Size	Small	Small	Big	Small
Detects Speed	Poor	Very Good	Good	Poor
Resolution	Poor	Average	Good	Very Good
Detects Distance	Good	Very Good	Good	Poor
Interference Susceptibility	Good	Poor	Good	Very Good
Field of View	Poor	Average	Very Good	Good
Accuracy	Poor	Average	Very Good	Very Good
Frame Rate	Average	Average	Average	Good
Colour Perception	Poor	Poor	Poor	Very Good
Maintenance	Average	Poor	Poor	Average
Visibility	Poor	Poor	Average	Good
Price	Good	Average	Poor	Average

**Table 2:** Hyperparameters used for the case study.

Hyperparameter	Value
Learning Rate	0.0001
Learning Rate Decay	1
GAE Discount Factor	0.99
GAE Lambda	0.95
Value Loss Scale Factor	1
Initial Deviation	0.7
Entropy Scale	0.01
PPO Epsilon	0.2
Horizon Number	32,768
Batch Size	2048
Epoch Number	4

**Table 3:** Curriculum steps for scenario 1.

Step	Vehicle	Restart	GNSS Error	Blackout	Town
1	Single	No	High	None	01
2	Single	No	High	None	02
3	Single	Yes	High	None	01/02
4	Single	Yes	High	None	02
5	Single	Yes	High	None	02
6	Single	Yes	Low	None	02

**Table 4:** Curriculum steps for scenario 2.

Step	Vehicle	Restart	GNSS Error	Blackout	Town
1	Single	No	High	None	01
2	Single	No	High	None	02
3	Single	Yes	High	None	01/02
4	Single	Yes	High	None	02
5	Single	Yes	High	GNSS	02
6	Single	Yes	Low	GNSS	02

**Table 5:** Curriculum steps for scenario 3.

Step	Vehicle	Restart	GNSS Error	Blackout	Town
1	Single	No	High	None	01
2	Single	No	High	None	02
3	Single	Yes	High	None	01/02
4	Single	Yes	High	None	02
5	Single	Yes	High	IMU/SAS/WO	02
6	Single	Yes	Low	IMU/SAS/WO	02

**Table 6:** Curriculum steps for scenario 4.

Step	Vehicle	Restart	GNSS Error	Blackout	Town
1	Single	No	High	None	01
2	Single	No	High	None	02
3	Single	Yes	High	None	01/02
4	Single	Yes	High	None	01
5	Single	Yes	Low	None	01

## Data Availability

Can be found in https://github.com/tulioaraujoMG/CarAware, accessed on 20 December 2025.
